# Increasing risks for emerging infectious diseases within a rapidly changing High Asia

**DOI:** 10.1007/s13280-021-01599-7

**Published:** 2021-07-22

**Authors:** Charudutt Mishra, Gustaf Samelius, Munib Khanyari, Prashanth Nuggehalli Srinivas, Matthew Low, Carol Esson, Suri Venkatachalam, Örjan Johansson

**Affiliations:** 1 Snow Leopard Trust, 4649 Sunnyside Avenue North, Seattle, USA; 2 Nature Conservation Foundation, 3076/5, IV Cross Gokulam Park, Mysore, India; 3 Nordens Ark, Åby Säteri, 456 93 Hunnebostrand, Sweden; 4 Interdisciplinary Center for Conservation Sciences, Oxford, University UK; 5 Department of Biological Sciences, University of Bristol, Bristol, UK; 6 Health Equity Cluster, Institute of Public Health, Bengaluru, India; 7 Department of Ecology, Swedish University of Agricultural Sciences, 75007 Uppsala, Sweden; 8 41 Walnut Close, Speewah, Queensland, 4881 Australia; 9 Department of Ecology, Grimsö Wildlife Research Station, Swedish University of Agricultural Sciences, 73091 Riddarhyttan, Sweden

**Keywords:** Mountains, One Health, Pandemics, *Panthera uncia*, Snow leopard, Zoonoses

## Abstract

The cold and arid mountains and plateaus of High Asia, inhabited by a relatively sparse human population, a high density of livestock, and wildlife such as the iconic snow leopard *Panthera uncia*, are usually considered low risk for disease outbreaks. However, based on current knowledge about drivers of disease emergence, we show that High Asia is rapidly developing conditions that favor increased emergence of infectious diseases and zoonoses. This is because of the existing prevalence of potentially serious pathogens in the system; intensifying environmental degradation; rapid changes in local ecological, socio-ecological, and socio-economic factors; and global risk intensifiers such as climate change and globalization. To better understand and manage the risks posed by diseases to humans, livestock, and wildlife, there is an urgent need for establishing a disease surveillance system and improving human and animal health care. Public health must be integrated with conservation programs, more ecologically sustainable development efforts and long-term disease surveillance.

**Supplementary Information:**

The online version contains supplementary material available at 10.1007/s13280-021-01599-7.

## Globally intensifying risk of disease emergence

**Fig. 1:**
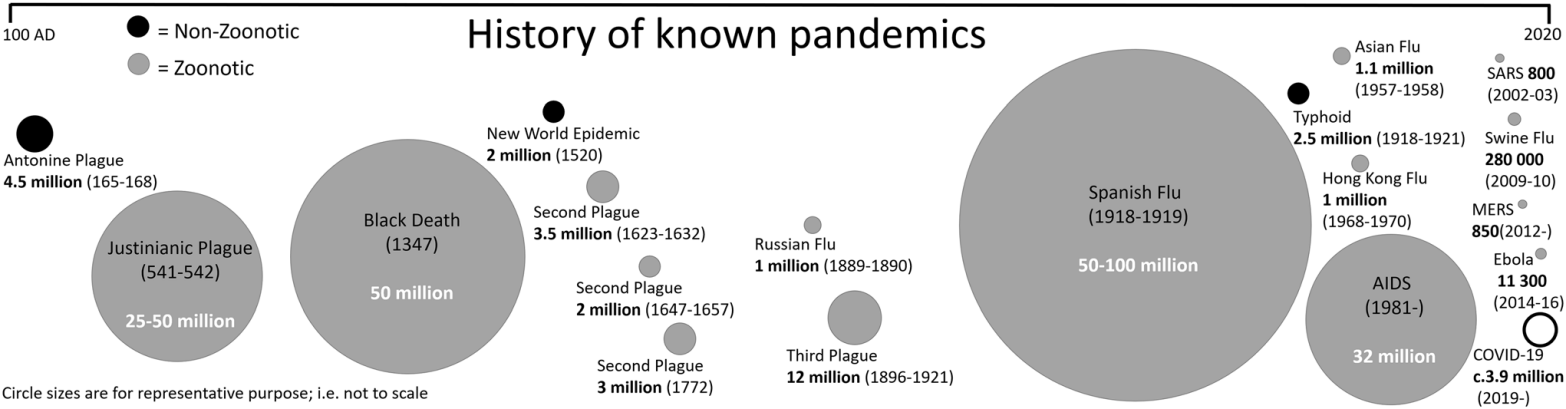
Known historical zoonotic (gray) and non-zoonotic (black) pandemics and associated human fatalities. The ongoing COVID-19 pandemic (shown as open circle) is thought to be of zoonotic origin. See Table S1 for details

Despite more than a century of progress in disease control, emerging infectious diseases (EIDs) are a growing problem for humans, wildlife, and domesticated species (Daszak et al. 2000). An estimated 75% of all EIDs are zoonotic, i.e., transmitted to humans from animals (Jones et al. 2008). They include recent coronavirus outbreaks in humans such as SARS and COVID-19, as well as other well-known diseases in human history including Ebola, HIV/AIDS, avian influenza, Lyme disease, and bubonic plague (Fig. 1). In addition to the obvious seriousness of many EIDs and the need to control outbreaks, their increasing global frequency (Allen et al. 2017; Fig. 1; Table S1) points to causative agents that are related to anthropogenic disturbance of ecosystems, biodiversity loss, and changes in the way people and animals interact (Daszak et al. 2000; Jones et al. 2008; Keesing et al. 2010). Consequently, increasing disease outbreaks could be expected in populations and regions that have previously been considered at low risk, especially as traditional societal structures, modes of agriculture, and natural environmental processes are disrupted (Lindahl and Grace 2015).

Several characteristics of globalization are linked to incidences of EIDs: (i) a highly integrated global economy leading to unprecedented movement of people, animals, and goods—and, thereby, pathogens—between regions, (ii) ecological disruption and habitat fragmentation that increase contact between people, wildlife, and domestic species, and (iii) general characteristics of development such as biotic homogenization that reduces ecosystem resilience, and pesticide and antibiotic use, which can compromise immune responses and promote the emergence of resistant pathogens (Jones et al. 2008; Karesh et al. 2012; Kilpatrick and Randolph 2012). Additional factors that further increase the risk of disease transmission between animals and people include poaching, trade in wildlife (Rosen and Smith 2010; Li and Lu 2014), consumption of wildlife, and the use of wildlife in traditional medicine (Mainka and Mills 1995; Graham-Rowe 2011). Thus, in areas undergoing significant changes in the frequency and type of human–animal interactions, monitoring disease transmission dynamics becomes critical for human health and welfare, and for the protection of wildlife populations and domestic livestock.

**Fig. 2:**
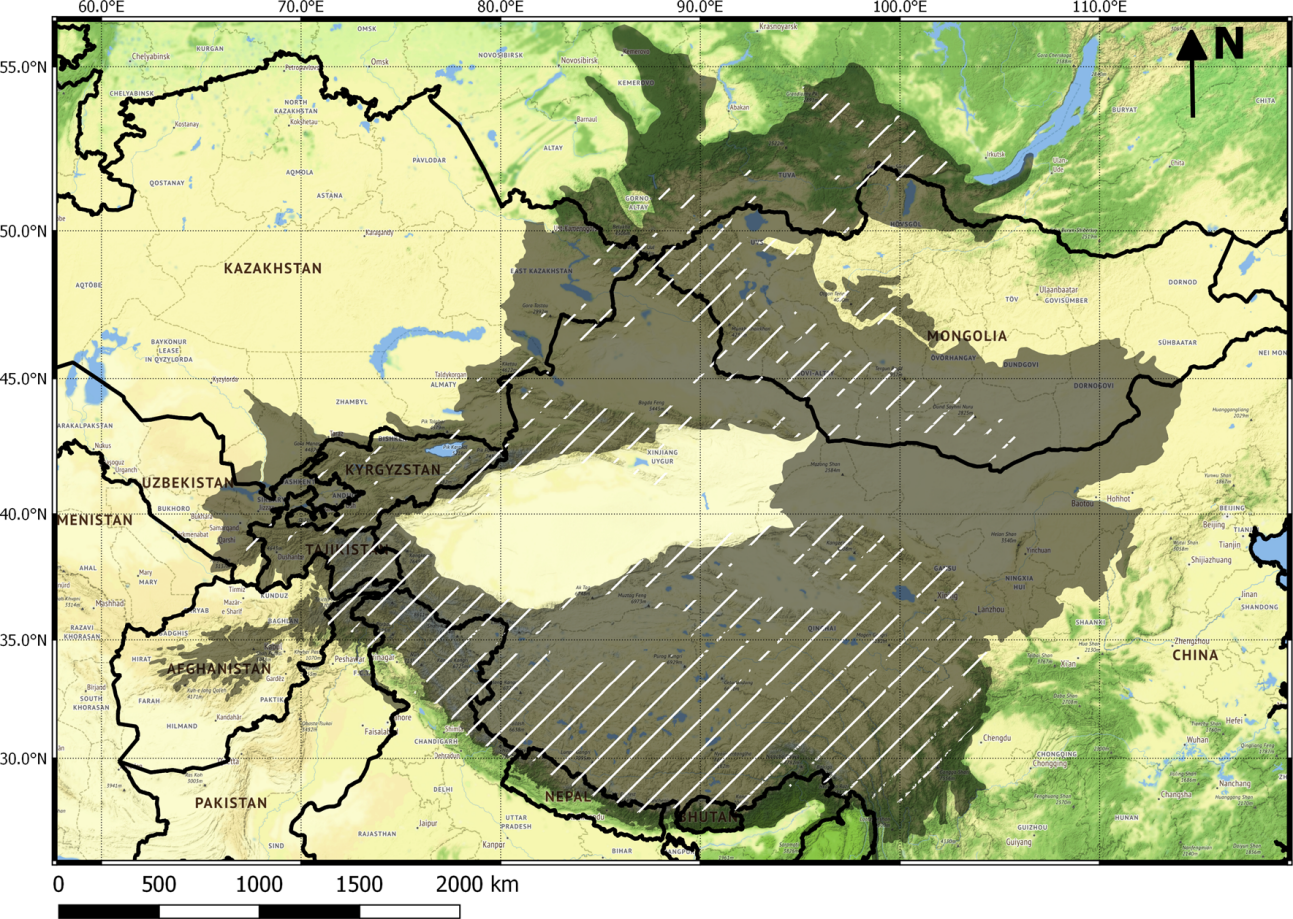
Map of High Asia (gray) including the estimated global range of the snow leopard (striped)

**Fig. 3:**
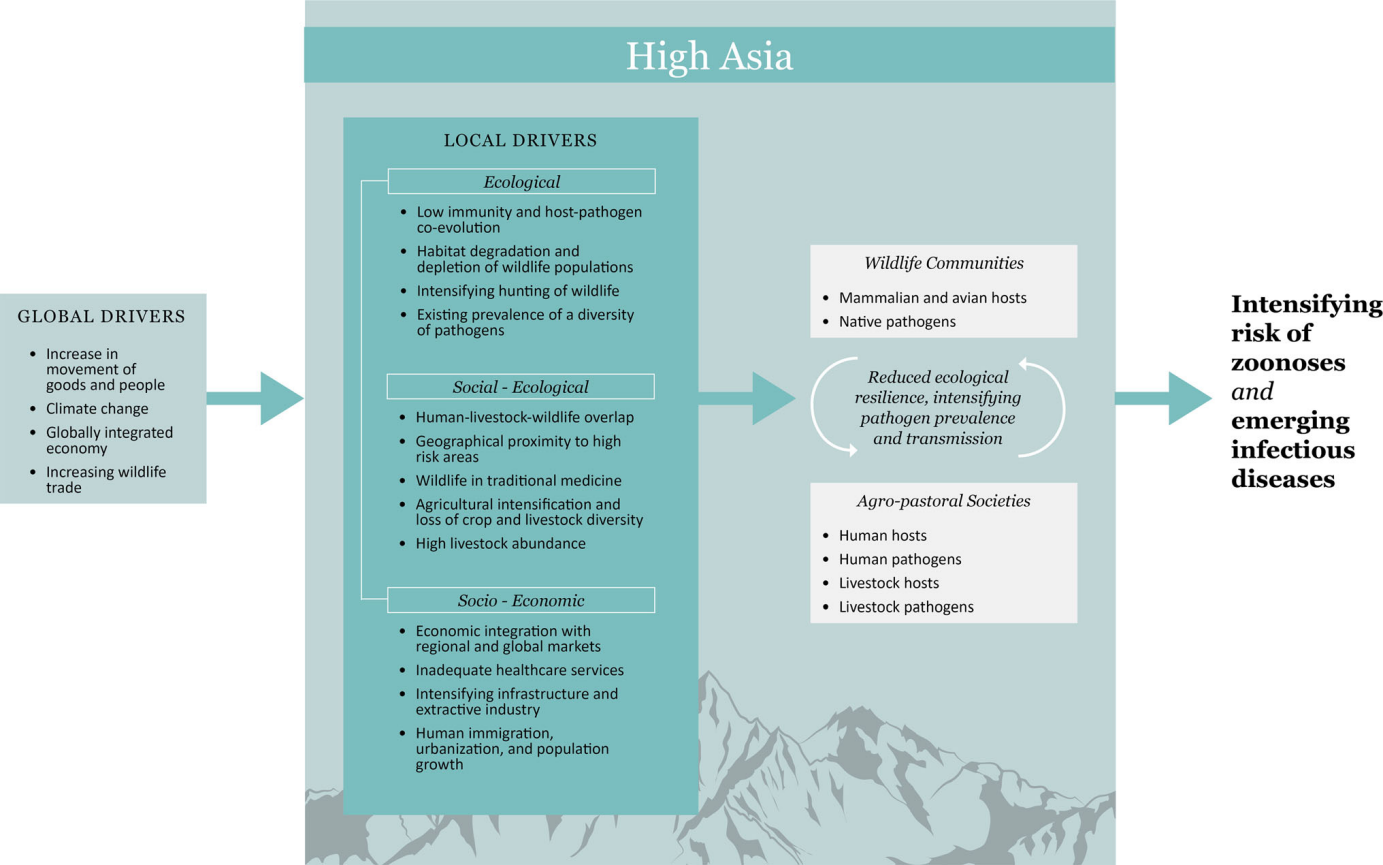
Schematic diagram illustrating the increasing risk of emerging infectious diseases and zoonoses due to changes in climate and socio-ecological dynamics in High Asia

Current forecast modeling indicates South and South East Asia, Central Europe, sub-Saharan Africa, Central America, and parts of South America as being at high risk of zoonotic outbreaks and EIDs (Jones et al. 2008; Allen et al. 2017). Naturally, these forecasts are based on past spillovers and outbreaks (Jones et al. 2008). Changing ecological and socio-economic conditions in areas previously considered as relatively ‘low risk,’ however, could tip the balance towards a higher risk category of EIDs. One such area is the contiguous region formed by the great mountain ranges, plateaus, and intersecting steppe and valleys of Asia, characterized primarily by the Central Asian dry alpine ecosystems: hereafter High Asia (Fig. 2, Table S2). High Asia is composed of ~ 6.4 million square kilometers and 44 ecoregions (Olson et al. 2001), and includes the Qinghai–Tibetan Plateau and parts of the great mountain ranges of the Himalayas, Pamirs, Karakoram, Tien Shan, and Altai (Table S2). Compared to tropical and temperate systems, High Asia has seen relatively little research on EIDs and zoonotic diseases (Saker et al. 2004; Allen et al. 2017). Here, we put forward the case that High Asia is rapidly developing conditions for increased risk of disease outbreaks in both human and animal populations. We collate evidence for the prevalence of various pathogens in this region and show how multiple ecological, socio-ecological, and socio-economic factors in High Asia may be interacting with climate change and globalization to create higher risk conditions for EIDs and zoonotic outbreaks compared to what current forecast modeling suggests (Fig. 3). We define High Asia to specifically include the distribution range of the apex mountain predator, the snow leopard (*Panthera uncia*), and the intervening regions connecting these mountains that are ecologically and climatically similar. We focus on the snow leopard range area because it typifies the human–livestock–wildlife relationships in this region, it is undergoing dramatic changes that we argue increase EID risk, and because of its importance for local cultural values and a globally significant wildlife conservation program. To define this region, we used the global ecoregion files for High Asia (published in Olson et al. 2001) and overlaid the current snow leopard distribution (Suryawanshi et al. 2019) using QGIS 2.18. Ecoregions coinciding with snow leopard distribution were included, as were interconnecting ecoregions that were identified by their sharing a border with at least one snow leopard ecoregion (these included mountainous ecoregions like intermontane steppe, mountain conifer forests, alpine meadows, and in limited cases non-mountainous ecoregions like desert steppes).

## Factors disposing High Asia to potential EIDs and zoonoses outbreaks

We consider the following local and global factors as likely to increase the risk of disease outbreaks in High Asia (Table 1; Fig. 3).

**Table 1:** Factors that reduce or increase the risk of transmission of emerging infectious diseases and zoonoses in snow leopard landscapes

Local factor	Lowering the risk	Intensifying the risk	References
Ecological factors			
Environmental conditions	Low temperatures, strong seasonality	Global warming	Li et al. (2016)
	Dry environment	Increasing precipitation and glacial melt	Turco et al. (2015)
Pathogens	Lower pathogen abundance	Low rates of immunity against pathogens	Ostrowski and Gilbert (2016)
Human population	Relatively low human density	Seasonal migration of humans and their livestock	Mishra et al. (2003b)
		Transition towards sedentarization	(Mishra et al. 2010)
Snow leopard	Low density	Large home ranges and movement distances	Johansson et al. (2016)
	High dependence on wild ungulates	Predation on livestock and retaliatory killing	Mishra et al. (2003a)
Other carnivores	Low densities	Close association between species	Pilot et al. (2018)
Wild ungulates	Low density	Large home ranges, migratory behavior	Haider et al. (2018); Joly et al. (2019)
		Hunting and consumption	Zahler et al. (2004)
		Taxonomically similar to livestock	Walker et al. (2017)
Birds		Large congregations of migrating birds	Prins and Namgail (2017)
		High proportion of migratory species	Marchenko et al. (2012)
Rodents	Relatively low abundance	Consumption of marmots for meat and medicine	Sariyeva et al. (2019)
		Relatively high densities of rodents in houses	Esson et al. (2019)
Socio-ecological factors			
Dogs		Increasing abundance of feral dogs in the landscape	Young et al. (2011)
Wildlife trade		Significant use of wild species in traditional medicinal systems	(Byard 2016)
Land use	Low intensity of land use	High abundance of livestock and close association with humans	Mijiddorj et al. (2018), Mishra et al. (2003b)
		High overlap in habitat use by livestock and wildlife	Mishra et al. (2004)
Socio-economic factors			
Human population		Increasing human density and urbanization	Mijiddorj et al. (2019), Murali et al. (2019)
Infrastructure and resource extraction		Increasing infrastructure development, mining, use of pesticides, and integration with lowlands	Snow Leopard Network (2014)
Wildlife trade		Demand for pelt, bones, and other tissues	Nowell et al. (2016)

### Local ecological factors

It is generally assumed that the cold and dry high-altitude landscapes of High Asia have lower abundance and richness of pathogens than the warmer lower elevations (Ostrowski and Gilbert 2016). However, the relative importance of pathogen abundance to the emergence of EIDs, compared to other risk factors, is largely unknown. It is also possible that lower intrinsic levels of immunity in animal populations because of lower rates of pathogen exposure or co-evolution of host–pathogen relationships compared to the tropics could render High Asian landscapes vulnerable to EIDs (Ostrowski and Gilbert 2016).

Recent studies of prominent wild animal taxa in High Asia, especially mammals, detail behavioral characteristics that could increase their disease exposure beyond what might be expected from the traditional view of these species (Table 1). A large proportion of High Asian mountains represent the global range of the snow leopard, a top predator and flagship species for biodiversity conservation (Fig. 2). Snow leopards occur at relatively low densities compared to other large felids (Karanth et al. 2004; Suryawanshi et al. 2019), where low densities are thought to limit the possibilities of encountering or spreading pathogens. However, snow leopards have been shown to have much larger home ranges (Johansson et al. 2016) and extensive movement behaviors (e.g., between mountain ranges [Johansson et al. 2020a]) than previously reported, increasing their chances of encountering and spreading disease within and across mountain ranges and international borders. Despite the belief that snow leopards are solitary outside mating (Fox and Chundawat 2016), individuals frequently interact, with adult males and females sometimes sharing kills and traveling together, as do females and their adult progeny (Johansson unpublished). Adults also frequently kill and interact with wild and domestic ungulate species (Johansson et al. 2015). From the perspective of snow leopard conservation, their population is likely to be relatively sensitive to disease impacts on mortality because of their slow reproductive rate related to late age at first breeding (3–4 years) and long inter-birth interval (minimum 24 months) when compared to other large felids (Johansson et al. 2020a).

The two most important prey species of the snow leopard, ibex (*Capra sibirica*) and blue sheep (*Pseudois nayaur*), show typically gregarious social behavior, along with seasonally compromised body condition due to strong seasonality and/or resource competition with sympatric livestock; factors important in regulating and spreading pathogens (Mishra et al. 2004; Ostrowski and Gilbert 2016). In addition, phylogenetic, dietary, and habitat similarities between wild ungulates and domestic livestock in this region can facilitate pathogen transfer between these groups. This has serious implications for disease risk, not only in wild and domestic animals, but also in the exposure risk of humans to these pathogens (Wolfe et al. 2007; Walker et al. 2017). Smaller species such as rodents and lagomorphs are known to carry over 60 zoonotic pathogens that can have serious effects on human and animal health (e.g., bubonic plague [Meerburg et al. 2009], Table 1). Rodents and lagomorphs are prey species of snow leopards (e.g., marmots *Marmota* spp. and hares *Lepus* spp. [Jumabay-Uulu et al. 2013]) and smaller rodents that live in close proximity to people and livestock in this region.

Bird migration is one risk factor contributing to the global spread of EIDs (Altizer et al. 2011; Reed et al. 2003). High Asia hosts breeding ground for a large number of birds, of which many are migratory (Prins and Namgail 2017). Two of the world’s eight main flyways for migratory birds, the Central Asia flyway and the East Africa West Asia flyway, cross High Asia (Olsen et al. 2006). Migratory birds traveling along these corridors move from areas densely populated by people and livestock in South-West Africa and Southern India, respectively, towards the northernmost part of Asia, thereby potentially transferring diseases between these populations. These migratory birds use High Asia stopover sites between their breeding and wintering areas, resulting in high seasonal bird concentrations at local water sources that are also used by resident wildlife, livestock, and local people (Krauss et al. 2010; Altizer et al. 2011).

### Local social–ecological factors

Human density in High Asia is relatively low, suggesting low risks of zoonotic EIDs. However, High Asian landscapes are used extensively for livestock production, and represent one of the largest rangeland systems in the world (Berger et al. 2013). The majority of people are agro-pastoralists and live in close proximity to livestock and wildlife (Mishra et al. 2003a, 2010), creating conditions for zoonotic spread; the abundance of livestock in this region is very high (Mishra et al. 2001; Berger et al. 2013); and people and their livestock migrate seasonally in many areas (Mijiddorj et al. 2018). These factors create a close connectivity between humans, livestock, and wildlife and their seasonal movements and interactions create a regional connectivity: key ingredients for the emergence and spread of disease (Daszak et al. 2000). Additionally, there is a considerable influx and egress of people through rapidly emerging tourism and other business and livelihood opportunities in High Asia (Mavroidi 2008; Mijiddorj et al. 2019). There is also a high dependence on traditional medicinal systems, many of which have a strong tradition of using body parts of wild species (Byard 2016; Negi and Palyal 2017), with markets where wildlife and medicines are traded in some parts of High Asia (Zhang et al. 2008).

Poaching and the trade of wildlife (both legal and illegal) is increasing in High Asia (Li and Lu 2014; Nowell et al. 2016) resulting in wildlife populations that are under significant threat (Table 1). This has implications for EIDs through both the increased direct contact of humans and wildlife and the wider ecosystem impacts associated with species-poor communities. In parts of the world, it is likely that hunting, handling, and consumption of wildlife have led to some of the most serious zoonotic outbreaks in recent times (e.g., HIV/AIDS, SARS, COVID-19). Disease outbreaks are also linked to the wider ecosystem effects that result from severely reduced wildlife populations, e.g., African trypanosomiasis is thought to have resulted from tsetse flies (*Glossina* spp.) switching from depleted wildebeest (*Connochaetes* spp.) populations to feed on humans (Karesh et al. 2012; WHO 2020). Similarly, the West Nile virus shows increased prevalence in species-poor bird communities, and the transmission risk of hantavirus to humans is related to a lower diversity of small mammals in the local environment (Keesing et al. 2010). Thus, state-supported eradication programs for rodents, lagomorphs, and even large carnivores such as wolves (*Canis lupus*) in High Asia (Kaczensky et al. 2008; Wilson and Smith 2015; Wu and Wang 2017) that might be expected to reduce the risk of EIDs by reducing the number of potential reservoirs, vectors, and encounter rate, in some cases may intensify the risk of disease outbreaks through a disruption of natural ecosystem interactions that increases the movement and turnover of animals in the system: factors known to increase disease spread (Epstein et al. 2006).

Agricultural practices in High Asia are currently changing through intensification and homogenization that intensify the likelihood of disease amplification and transmission (Epstein et al. 2006): new areas are being brought under cropland (Namgail et al. 2007), there is reduction of crop and livestock diversity (Mishra et al. 2003b) and an increase in livestock densities (Berger et al. 2013). Modern livestock management and its interface with wildlife can result in increased cycling of bacterial strains that carry antibiotic resistance (Vittecoq et al. 2016), which can then be acquired and transported by wildlife, with omnivorous, anthropophilic, and carnivorous species being at higher risk because of their close interactions with livestock in this region (Arnold et al. 2016).

### Local socio-economic factors

Rapid urbanization and population growth are currently occurring in High Asia (Tiwari et al. 2018; Murali et al. 2019). The 12 countries that form the global range of the snow leopard account for 42.6% of the world’s human population (World Bank 2020). Many of these countries also encompass temperate and tropical zones that represent the geographical origins of several major known EIDs (e.g., bubonic plague, COVID-19, SARS, and Kyasanur Forest Disease (Bordier and Roger 2013)) and are currently considered as high risk areas. The local economies and production systems in High Asia are increasingly integrated with national economies, resulting in a high movement of goods and people between the mountains and low-lying areas (Mishra 2000; Berger et al. 2013; Snow Leopard Working Secretariat 2013). This includes a rapidly expanding tourism industry (Mavroidi 2008; Alexander et al. 2019; Mijiddorj et al. 2019). Large parts of High Asia are remote which results in law enforcement being difficult, increasing the possibility of illegal hunting of wildlife for consumption and trade (Mishra and Fitzherbert 2004; Zahler et al. 2004).

The number of feral dogs (*Canis familiaris*) in many areas of High Asia is increasing as a consequence of urbanization in countries such as Bhutan, China, India, Nepal, Pakistan, and Uzbekistan (Snow Leopard Network 2014). Feral and herding dogs also increase the links between remote habitats and urban centers, and facilitate pathogen transmission between wild species, livestock, and humans (Gascoyne et al. 1993; Randall et al. 2004; Budke et al. 2005).

Finally, High Asian landscapes form smaller, fringe parts of most of the constituent countries and are often considered remote and atypical. Hence, their national public services—including public and animal health—are not specifically adapted to the local conditions. In addition, inadequate investments in health care services and disease surveillance in High Asia limit the ability to predict or prepare for possible outbreaks on both national and regional scales.

### Global factors

High Asia is currently experiencing rapid transitions linked to globalization and climate change (Table 1). These changes have strong implications for increasing the risk of EIDs and zoonoses through introduction of new pathogens and vectors, habitat fragmentation, pollution, and human migration (Lerer and Scudder 1999; Patz et al. 2004).

The mountains and plateaus of High Asia are among the world’s most vulnerable areas from a climate change perspective, warming at more than twice the average rate of the northern hemisphere (Li et al. 2016). Climate change will affect parasite–host assemblages, and likely result in increased frequency and intensification of disease outbreaks (Harvell et al. 2002; Brooks and Hoberg 2007; Fig. 3). In 2015, there was a mass mortality event of around 200 000 saiga (*Saiga tatarica*) (65% of the global population) due to hemorrhagic septicemia caused by the bacterium (*Pasteurella multocida*), which although outside the region we are describing, exemplifies the reality of changing climate influencing parasite–host interactions in ways which may lead to serious disease outbreaks and higher rates of mortality (Rohr et al. 2013; Kock et al. 2018).

The once remote landscapes of High Asia are now under considerable pressures of economic and infrastructure development, resulting in the opening up of remote habitats and increasing integration with lowlands and mainstream economies (Snow Leopard Working Secretariat 2013; Snow Leopard Network 2014;). These infrastructure developments include mining, gas and oil pipelines, new roads and railways, and large dams (Grumbine and Pandit 2013; Snow Leopard Network 2014; Ascensão et al. 2018). Such large development projects are associated with pollution and immigration of workers from outside areas, opening up new markets for livestock and wildlife trade, and introducing foreign pathogens and vectors (Kilpatrick and Randolph 2012; Hassell et al. 2017). There are also unprecedented levels of long-distance movement of people and goods between High Asia and the rest of the world, creating strong pathways for the spread of disease both into and out of the region (Berger et al. 2013).

## Pathogen prevalence in High Asia

Knowledge of pathogen prevalence and disease transmission within and between wildlife and domestic animal populations of High Asia is limited and scattered. Nevertheless, it is evident from the available information that many virulent pathogens with the potential to cause widespread mortality and morbidity, in both animals and people, already exist in the animal populations in High Asia (Table 2). These pathogens and potential disease outbreaks have serious implications for both the health of people and their livelihoods and the conservation of wildlife not just in High Asia, but they also pose a global challenge (Daszak et al. 2000).

**Table 2:** Main zoonoses in High Asia sorted by type of disease (viral, bacterial, and parasitic) and main hosts involved

Disease	Pathogen	Main hosts	Mode of transmission	References
Viruses				
Rabies	*Rabies virus*	Carnivores and bats	Bites and saliva	Ebright et al. (2003)
SARS	SARS-coronavirus	Bats and possibly small carnivores	Airborne droplets	Bell et al. (2004); Li et al. (2005); Ye et al. (2020)
MERS	MERS-coronavirus	Bats and possibly camels	Airborne droplets	Ramadan and Shaib (2019); Ye et al. (2020)
COVID-19	SARS-coronavirus 2	Likely bats	Airborne droplets	Malik et al. (2020); Ye et al. 2020)
Avian influenza	Influenza A virus subtype H5N1	Birds		Marchenko et al. (2012)
Swine influenza	Influenza A virus subtype H1N1	Pigs	Airborne droplets	Kothalawala et al. (2006)
Hantavirus	Hantavirus	Rodents	Airborne transmission	Bi et al. (2008)
Japanese encephalitis	Japanese encephalitis virus	Birds and possibly pigs	Vectors	Ladreyt et al. (2019)
Influenza	Influenza A virus	Birds	Airborne droplets	Webby et al. (2007)
Bacteria				
Anthrax	*Bacillus anthracis*	Ungulates	Inhalation and ingestion	Ebright et al. (2003)
Plague	*Yersinia pestis*	Rodents	Arthropod vectors	Alfani and Murphy (2017); Ebright et al. (2003)
Tuberculosis	*Mycobacterium* spp.	Birds, mammals, and reptiles	Airborne droplets and aerosols	De Lisle et al. (2002)
Brucellosis	Brucella spp.	Ungulates	Ingestion	Ebright et al. (2003)
Leptospirosis	*Leptospira* spp.	Rodents and other mammals	Ingestion, broken skin	Victoriano et al. (2009)
Tularemia	*Francisella tularensis*	Mammals and birds	Vectors, ingestion, and air	Keim et al. (2007)
Cat-scratch disease	*Bartonella* spp.	Rodents and felids	Vectors	Chomel and Kasten (2010)
Q fever	*Coxiella burnetii*	Ungulates and other mammals	Airborne aerosols	Gonzalez-Barrio and Ruiz-Fons (2019)
Lyme disease	*Borrelia burgdorferi*	Rodents and ungulates	Arthropod vectors	Anderson (1988); Levi et al. (2012)
Parasites				
Echinococcosis	*Echinococcus* spp.	Canids, rodents, and ungulates	Ingestion	Otero-Abad and Torgerson (2013)
Trichinosis	*Trichinella* spp.	Pigs and carnivores	Ingestion	Kagan (1960)
Giardiasis	*Giardia duodenalis*	Mammals and birds	Ingestion	Appelbee et al. (2005)
Toxoplasmosis	*Toxoplasma gondii*	Mammals and birds	Ingestion	Dubey (2010)
Cryptosporidiosis	*Cryptosporidium* spp.	Mammals and birds	Ingestion	Appelbee et al. (2005)

Included in the table is also the main mode of transmission

### Snow leopards

Research on disease in wild snow leopards has been very limited and most information to date comes from captive animals. Our work in Tost Mountains of Mongolia discovered antibodies to several zoonotic pathogens in wild snow leopards (Esson et al. 2019). Other pathogens from the study in Mongolia included *Coxiella burnetii*, the sublethal but highly virulent bacterium often found in livestock that causes Q fever in humans; the protozoan parasite *Toxoplasma gondii* that causes Toxoplasmosis; and bacteria belonging to the genus *Leptospira* that cause potentially life-threatening Leptospirosis in humans (Esson et al. 2019). In the same study, we recorded several zoonotic bacteria from ectoparasitic ticks collected from snow leopards belonging to the genera *Bacillus, Bacteroides, Campylobacter, Coxiella, Rickettsia, Staphylococcus,* and *Streptococcus*. Bacteria within these genera are responsible for severe illnesses in humans including anaplasmosis, Q fever, ehrlichiosis, and anthrax. Among other common bacterial zoonosis, tuberculosis has been reported in captive snow leopards but there are no reports of plague or anthrax in either wild or captive snow leopards (Helman et al. 1998). However, confirmed existence of plague and related zoonosis from marmots, anthrax in wild ungulate prey of snow leopards, and the fact that felids like mountain lions *Puma concolor* act as vectors for plague and anthrax, render them a potentially serious concern for snow leopards (Hugh-Jones and de Vos 2002). There are several viral diseases reported in captive snow leopards that are typically specific to felines such as feline coronavirus, feline parvovirus, calicivirus, feline immunodeficiency virus, feline panleukopenia, feline papillomavirus, canine distemper virus, and papillomavirus (Kennedy et al. 2002; Mitsouras et al. 2011; Ostrowski and Gilbert 2016). COVID-19 was also reported in three captive snow leopards in USA (Andrew 2020). The only study on viral diseases in free-ranging snow leopards to date found that four of six sampled snow leopards had been infected by rotavirus. The study also reported infections of felid herpesvirus 1 and feline papillomavirus 2 (Johansson et al. 2020b). Snow leopards from the same study area also carried antibodies against feline corona virus (Snow Leopard Trust, unpublished data). While there are no reports of snow leopards dying from any of these diseases, felids in other parts of the world are known to die from diseases such as canine distemper virus, rendering diseases a potentially serious concern in snow leopard landscapes (Roelke-Parker et al. 1997).

### Canids

Both wild and domestic canids in High Asia carry important zoonotic diseases, and since herding and feral dogs interact with people, livestock, and wildlife, they are an important potential route for disease transmission as occurred with rabies outbreaks in African wild dogs (*Lycaon pictus*) and Ethiopian wolves (*Canis simensis*) (Gascoyne et al. 1993; Randall et al. 2004). Rabies has been reported in foxes, dogs, and wolves in High Asia (Boldbaatar et al. 2010), but its current impacts and future emergence potential are uncertain. Echinococcosis, a zoonotic infection caused by tapeworms from the genus *Echinococcus* has seen a recent re-emergence in Central Asia (Torgerson et al. 2006; Ziadinov et al. 2008), with the parasite largely prevalent in feral and herding dogs, and red foxes (*Vulpes Vulpes*) acting as reservoirs (Kruse et al. 2004).

### Ungulates

Potentially zoonotic diseases such as sarcoptic mange and mycoplasmosis have caused mortalities of ungulates including blue sheep (*Pseudois nayaur*) and markhor (*Capra falconeri*) in the mountains of Pakistan and Tajikistan, respectively. These outbreaks are linked to transfer from sympatric livestock (Dagleish et al. 2007; Ostrowski et al. 2011). Bubonic plague (*Yersinia pestis*) and anthrax (*Bacillus anthracis*) are also carried by wild ungulates and smaller prey taken by snow leopards, such as marmots (*Marmota baibacina*) (Sariyeva et al. 2019). Marmots are hunted by people for their meat in large parts of the snow leopard range, and associated human deaths from plague transmission have been reported recently (Grewal 2020).

### Bats

Research on bat distribution and related zoonotic diseases has been limited across High Asia (Mackenzie and Williams 2009; Gay et al. 2014), despite them being common and known to carry some of the most serious zoonotic diseases in the world (Kuzmin et al. 2006; Luis et al. 2013). Kuzmin et al. (2006) isolated rabies virus from two different bat species from parts of High Asia, and species of horseshoe bats (e.g., *Rhinolophus ferrumequinum*, *R. hipposideros*, and *R. sinicus*), known to host various types of coronaviruses (including SARS and COVID-19) are also present (Hu et al. 2017; Zhou et al. 2020); albeit with lower abundance than most low-lying areas. The presence of *Anaplasma* bacterial infection in ticks from the Qinghai–Tibetan plateau has been confirmed with bats being suggested as potential hosts (Han et al. 2019). In the relative cold and harsh environment of High Asia, many bat species hibernate in large groups through the winter (Geiser and Turbill 2009), increasing the risk of disease transmission. Similarly, current anthropogenic habitat modifications are likely to increase exposure to, and drive behavior-related changes in bats that increase the risk of disease transmission to humans, livestock, and other wildlife (Hayman et al. 2013).

### Birds

Avian influenza is a zoonotic EID that can cause disease symptoms in birds and people that range from mild respiratory symptoms to acute respiratory distress and death (Hu et al. 2011; Marchenko et al. 2012). A large outbreak of H5N1 influenza virus occurred in Qinghai lake, China in 2005 and viruses from the same clade were discovered in Mongolia, Russia, Europe, and Africa along the birds’ migratory flyways. Since then, several outbreaks of avian influenza have occurred in the snow leopard distribution area (Hu et al. 2011). Marchenko et al. (2012) sampled birds belonging to six orders across Kazakhstan, Mongolia, and Russia and found six isolates of the avian influenza virus in Mongolia and Russia. The risk of zoonoses and EIDs from migratory bird populations in High Asia remains significant and is likely increasing.

## Conclusions and the way ahead

The cold and arid mountains and plateaus of High Asia have so far been considered at low risk of disease emergence, and thus, relatively neglected from the perspective of disease research, and their potential implications for human health and biodiversity conservation (Jones et al. 2008; Allen et al. 2017). However, a closer examination of the region shows that with ongoing changes in ecological, socio-ecological, and socio-economic factors, there is an increased potential for EID outbreaks that could have large impact on local communities and biodiversity conservation (Fig. 3). Increased movement of people and livestock along the regional 'Belt and Road Initiative’ is projected to increase the risk of diseases through several countries within High Asia (Hughes et al. 2020). Global factors including climate change and globalization further intensify this risk (Lerer and Scudder 1999; Patz et al. 2004) and as has been demonstrated well by recent pandemics, local disease outbreaks now have the potential to threaten humans and animals around the world due to globalization (Daszak et al. 2000).

Thus, there is an urgent need for inter-sectoral (human and animal health, agriculture, and conservation) and multi-disciplinary engagement in setting up long-term disease surveillance programs across High Asia. These programs need to not only focus on EIDs and zoonoses of current concern, but also engage in long-term strategic monitoring of endemic diseases within wildlife and domestic livestock to help understand how interactions among these pools may affect each other, and to help understand where new EIDs may arise. This needs to be accompanied by a systematic strengthening of health systems in these remote areas to ensure appropriate health care and well-being of local communities, which in turn, will indirectly provide wider benefits at the national and global levels by earlier detection and preparedness for EIDs and zoonoses (Daszak et al. 2000; Al-Kindi 2020). Currently, most surveillance in many low- and middle-income countries occurs is in isolation, with limited data-sharing and lack of integrated responses during outbreaks (Chatterjee et al. 2016). Thus, wide-scale changes in this area are urgently needed, for example, recent policy recommendations in India (National Mission on Biodiversity and Human Well-being), which aim to work towards a healthier and more sustainable way of life, for people and nature (Bawa et al. 2020).

We suggest that research and management of potential EIDs and zoonoses must become a priority for human welfare and biodiversity conservation in High Asia. Spatially explicit monitoring of factors that increase the risk of disease outbreaks is necessary (Daszak et al. 2000). This is especially important today when climate change and increasing globalization are favoring EIDs and zoonoses (Harvell et al. 2002; Jones et al. 2008). Moreover, the growing rate of poaching and illegal wildlife trade needs to be disrupted through stronger law enforcement, and better cooperation among governments, conservationists, and local communities (Cooney et al. 2017). We also call for raising awareness among local and global communities about the threat of EIDs and zoonotic diseases and discourage the use of wild animal products (Daszak et al. 2000). Better veterinary care of livestock and stronger training of vets in wildlife diseases is also needed. Controlling feral dog populations where they have become a conservation management problem as well as human health hazard is important as well (Young et al. 2011).

There is great need and opportunity to enable sustainable economies that maintain the integrity and resilience of natural ecosystems of High Asia, which, in addition to improving human well-being, can help maintain systemic resilience against disease emergence. Promoting green infrastructure, organic farming, and green economies that minimize ecosystem damage, while supporting enterprises that are dependent on ecosystem services can help ensure a healthy and sustainable future for humans and biodiversity in High Asia, with positive global consequences.

As we have suggested in this review, and as the world has witnessed in the outbreaks of zoonoses such as SARS and COVID-19, concerns of human and environmental health and the well-being of people and ecosystems are intricately linked and global in nature (the ‘One Health’ concept; Harvell et al. 2002; Jones et al. 2008). Human health and well-being and the well-being of ecosystems should not be treated as separate policy realms, or viewed as local health or conservation problems, but instead be viewed and managed as integrated entities (Daszak et al. 2000). We therefore reiterate that there is need for holistic development of sustainable health, economic, and biodiversity conservation systems, particularly, in High Asia and other regions with similar settings and changes. With its significant geographical extent, often healthy ecosystems, clean water resources, and other ecosystem services, High Asia offers great opportunities to contribute sustainably towards better planetary health (Whitmee et al. 2015). The high-level inter-governmental alliance of 12 countries focused on snow leopard conservation in High Asia, called the Global Snow Leopard and Ecosystem Protection Program (Snow Leopard Working Secretariat 2013), can play a pivotal role in prioritizing human and wildlife health issues and addressing these various needs.

## Supplementary Information

Below is the link to the electronic supplementary material.

Supplementary file1 (PDF 569 KB)
