# Supplementary material for: Increasing risks for emerging infectious diseases within a rapidly changing High Asia
Source: Ambio. 2021 Jul 22;51(3):494–507. doi: 10.1007/s13280-021-01599-7 (PMC8297435; doi:10.1007/s13280-021-01599-7)
Supplement: Supplementary file 1 — Supplementary file1 (PDF 569 KB) [file 13280_2021_1599_MOESM1_ESM.pdf]

Ambio

Electronic Supplementary Material
This supplementary material has not been peer reviewed.

Title: **Increasing risks for emerging infectious diseases within a rapidly changing High Asia**

Authors: Charudutt Mishra, Gustaf Samelius, Munib Khanyari, Prashanth Nuggehalli Srinivas, Matthew Low, Carol Esson, Suri Venkatachalam, Örjan Johansson

Table S1. History of known pandemics where zoonotic pandemics are in black text and non-zoonotic pandemics in blue text. Abbreviations for the sources are WHO for the World Health Organization and CDC for the Centers for Disease Control and Prevention.

Time period	Pandemic	Pathogen	Number of human fatalities	Source
165-168	Antonine Plague	Likely smallpox	3.5-5 million	Littman and Littman (1973)
541-542	Justinianic Plague	*Yersinia pestis*	25-50 million	Alfani and Murphy (2017)
1347-1351	Black Death	*Yersinia pestis*	50 million	Alfani and Murphy (2017)
1520	New World Epidemic	Smallpox	2 million*	Anderson et al (1986)
1623-1632	Second Plague	*Yersinia pestis*	3.5 million	Alfani and Murphy (2017)
1647-1657	Second Plague	*Yersinia pestis*	2 million	Alfani and Murphy (2017)
1772	Second Plague	*Yersinia pestis*	2-4 million	Bramanti et al (2016), Shahriki et al (2016)
1889-1990	Russian Flu	Likely influenza A virus subtype H3N8	1 million	Valleron et al (2010)
1896-1921	Third Plague	*Yersinia pestis*	12 million	Alfani and Murphy (2017)
1918-1919	Spanish Flu	Influenza A virus subtype H1N1	50-100 million	Morens and Fauci (2007), CDC
1918-1921	Typhoid	*Rickettsia prowazekii*	2.5 million	Anderson et al (1986)
1957-1958	Asian Flu (1957-1958 Pandemic)	Influenza A virus subtype H2N2	1.1 million	Vibound et al (2016), CDC

1968-1970	Hong Kong Flu (1968 Pandemic)	Influenza A virus subtype H3N2	1 million	CDC
1981-present	AIDS	Lentivirus	32 million	WHO
2002-2003	SARS	SARS-coronavirus 1	800	CDC
2009-2010	Swine Flu (2009 Pandemic)	Influenza A virus subtype H1N1	280,000	Dawood et al (2012), CDC
2012-present	MERS	MERS-coronavirus	850	WHO
2014-2016	Ebola	Ebola-virus	11,300	WHO, CDC
2019-present	COVID-19	SARS-coronavirus 2	3,900,000 as of 22 Jun 2021	WHO

Table S2. Ecoregions that comprise High Asia.

Sr. no.	Eco regions	Area (km^2)
1	Afghan Mountains semi-desert	13682
2	Alai-Western Tian Shan steppe	127683
3	Alashan Plateau semi-desert	674352
4	Altai alpine meadow and tundra	90434
5	Altai montane forest and forest steppe	142875
6	Altai steppe and semi-desert	83192
7	Central Tibetan plateau alpine steppe	629190
8	Eastern Gobi desert steppe	282368
9	Eastern Himalayan alpine shrub and meadows	121014
10	Eastern Himalayan Broadleaf forests	82916
11	Eastern Himalayan sub-alpine coniferous forests	27436
12	Emin Valley Steppe	65135
13	Ghorat-Hozarat Alpine meadow	66482
14	Gissaro-Alai open woodlands	168170
15	Gobi Lakes Valley desert steppe	139714
16	Great Lakes Basin desert steppe	157706
17	Hengduan Mountains subalpine conifer forests	99291
18	Helanshan Montane conifer forests	24704
19	Hindu Kush alpine meadow	28268
20	Junggar Basin semi-desert	304938
21	Karakoram-West Tibetan Plateau alpine steppe	143265

22	Khangai Mountains alpine meadow	37168
23	Khangai Mountains conifer forests	2902
24	North Tibetan Plateau-Kunlun Mountains alpine desert	374494
25	Northeastern Himalayan subalpine conifer forests	46220
26	Northwestern Himalayan alpine shrub and meadows	49390
27	Nujiang Langcang Gorge alpine conifer and mixed forests	82699
28	Ordos Plateau steppe	215604
29	Pamir alpine desert and tundra	118072
30	Qaidam Basin semi-desert	192147
31	Qilian Mountains conifer forests	16653
32	Qilian Mountains subalpine meadows	73285
33	Qionglai-Minshan conifer forest	80134
34	Rock and Ice	0
35	Sayan Alpine meadows and tundra	81213
36	Sayan Intermontane steppe	34057
37	Sayan montane conifer forests	358833
38	Southeast Tibet shrublands and meadows	460542
39	Tian Shan foothill arid steppe	129231
40	Tian Shan montane conifer forests	27568
41	Tian Shan montane steppe and meadows	280611
42	Tibetan Plateau alpine shrublands and meadows	271999
43	Western Himalayan alpine shrub and Meadows	70090
44	Western Himalayan subalpine conifer forests	39650
45	Yarlung Tsangpo arid steppe	59381

Olson, D. M., Dinerstein, E., Wikramanayake, E. D., Burgess, N. D., Powell, G. V. N., Underwood, E. C., D'Amico, J. A., Itoua, I., Strand, H. E., Morrison, J. C., Loucks, C. J., Allnutt, T. F., Ricketts, T. H., Kura, Y., Lamoreux, J. F., Wettengel, W. W., Hedao, P., Kassem, K. R. 2001. Terrestrial ecoregions of the world: a new map of life on Earth. Bioscience 51: 933-938.
